# Cognitive impairment and associated factors among patients with diabetes mellitus in Africa: a systematic review and meta-analysis

**DOI:** 10.3389/fendo.2024.1386600

**Published:** 2024-07-17

**Authors:** Worku Chekol Tassew, Yeshiwas Ayal Ferede, Agerie Mengistie Zeleke

**Affiliations:** ^1^ Department of Medical Nursing, Teda Health Science College, Gondar, Ethiopia; ^2^ Department of Reproductive Health, Teda Health Science College, Gondar, Ethiopia; ^3^ Department of Clinical Midwifery, Teda Health Science College, Gondar, Ethiopia

**Keywords:** cognitive impairment, diabetes mellitus, systematic review, meta-analysis, Africa

## Abstract

**Background:**

Inappropriate management of blood sugar in patients with diabetes mellitus leads to micro-vascular and macro-vascular complications, subsequently leading to high morbidity and mortality rates. In addition, diabetes independently increases the occurrence of cognitive impairment complicated by dementia. Scientific evidence on the magnitude of cognitive impairment will provide a sound basis for the determination of healthcare needs and the planning of effective healthcare services. Despite this, there are no comprehensive data on the prevalence and associated factors of cognitive impairment among patients with diabetes in Africa.

**Methods:**

To identify relevant articles for this review, we searched PubMed, Cochrane Library, Science Direct, African Journals Online, and Google Scholar. After extraction, the data were imported into Stata software version 11 (Stata Corp., TX, USA) for further analysis. The random-effects model, specifically the DerSimonian and Laird (D+L) pooled estimation method, was used due to the high heterogeneity between the included articles. Begg’s and Egger’s regression tests were used to determine the evidence of publication bias. Sub-group analyses and sensitivity analyses were also conducted to handle heterogeneity.

**Results:**

The pooled prevalence of cognitive impairment among patients with diabetes in Africa is found to be 43.99% (95% CI: 30.15–57.83, *p* < 0.001). According to our analysis, primary level of education [pooled odds ratio (POR) = 6.08, 95% CI: 3.57–10.36, *I* ^2^ = 40.7%], poorly controlled diabetes mellitus (POR = 5.85, 95% CI: 1.64–20.92, *I* ^2^ = 87.8%), age above 60 years old (POR = 3.83, 95% 95% CI: 1.36–10.79, *I* ^2^ = 63.7%), and diabetes duration greater than 10 years (POR = 1.13; 95% CI: 1.07–1.19, *I* ^2^ = 0.0%) were factors associated with cognitive impairment among patients with diabetes.

**Conclusion:**

Based on our systematic review, individuals with diabetes mellitus exhibit a substantial prevalence rate (43.99%) of cognitive impairment. Cognitive impairment was found to be associated with factors such as primary level of education, poorly controlled diabetes mellitus, age above 60 years, and diabetes duration greater than 10 years. Developing suitable risk assessment tools is crucial to address uncontrolled hyperglycemia effectively.

**Systematic review registration:**

https://www.crd.york.ac.uk/prospero, identifier CRD42024561484.

## Introduction

Diabetes mellitus (DM) is a group of metabolic disorders characterized by elevated levels of glucose in the blood resulting from defects in insulin secretion, insulin action, or both (1). The burden of DM is increasing worldwide, especially in developing countries (2). In 2014, the global prevalence of diabetes was 422 million, and by 2040, this number is expected to rise to more than 642 million. The healthcare costs for DM reached 162 billion dollars in 2019 and will be 185 billion dollars in 2045 (3, 4). Inappropriate management of blood sugar in patients with DM leads to micro-vascular and macro-vascular complications, subsequently leading to high morbidity and mortality rates. In addition, diabetes independently increases the occurrence of cognitive impairment (CI), which is complicated by dementia (5–7).

CI is defined as a disturbance in memory, acquiring knowledge, focusing, or making decisions that have a negative impact on activities of daily life (8). Patients with DM are more likely to develop cognitive problems and dementia than patients without DM (9). DM can lead to the accumulation of waxy protein in the neuron by decreasing its excretion through cerebrospinal fluid, ultimately resulting in cognitive decline (10). Extended exposure of nerve cells to high levels of glucose weakens the connections between neurons, leading to their distortion, a condition directly linked to cognitive dysfunction (7).

Different studies have suggested that the exact pathophysiology of CI in diabetic patients may be due to blood vessel abnormality, insulin transmission disturbance in the cerebrum, recurrent attack of nerve cells with hyperglycemia and hypoglycemia, and accumulation of waxy protein in the neuron (11, 12). The global prevalence of CI is 45% (13) with the lowest and highest prevalence being 21.8% and 67.5%, respectively (14, 15). The global prevalence of complications of CI (dementia) in 2010 was 35.6 million, which is expected to rise to 65.7 million by 2030 (16). The overall prevalence of CI in sub-Saharan African countries among the general population has been estimated to be between 6.3% and 25% (17). The comorbidity of DM and CI is the major challenge for the long-term management of DM. DM not only causes CI but also induces the complications of CI (18). The global healthcare cost of CI complicated by dementia is 1.5 times higher than that of patients without dementia (19).

An increase in the magnitude of CI with the high cost of healthcare will impose a serious social, medical, and economic burden, causing a major challenge to the already strained healthcare system of African countries. CI is a major problem that affects the effective long-term management of diabetes. Early diagnosis of CI in patients with diabetes is important for the recovery of cognitive function and the delay of cognitive decline, as well as improving medication adherence in people with diabetes. Scientific evidence on the magnitude of CI will provide a sound basis for the determination of healthcare needs and the planning of effective healthcare services.

Despite this, there are no comprehensive data on the prevalence and associated factors of CI among patients with diabetes in Africa. We therefore designed this review to assess the prevalence and associated factors of CI among patients with diabetes in Africa.

## Materials and methods

The international Preferred Reporting Items for Systematic Reviews and Meta-Analysis (PRISMA) guidelines were used (20) to report the findings of this systematic review and meta-analysis ( **Supplementary Material 1** ).

### Publication search strategy

To identify pertinent articles for this review, we conducted searches in several databases, including PubMed, Cochrane Library, Science Direct, African Journals Online, and Google Scholar. These searches were performed by two authors (WCT and AMZ) between 10 November 2023 and 10 January 2024. We used the following MeSH terms while searching from the above electronic databases: “cognitive dysfunction” OR “cognitive impairment” OR “neurocognitive disorder” OR “cognitive decline” AND “diabetes” OR “diabetes mellitus” OR “diabetes mellitus type 2” OR “diabetes mellitus type II” OR “type II diabetes mellitus” OR “type 2 diabetes mellitus” OR “type 2 diabetes” OR “diabetes mellitus type 1” OR “diabetes mellitus type I” OR “type I diabetes mellitus” OR “type 1 diabetes mellitus” OR “type 1 diabetes”. The snowball technique from the searched articles was also used to obtain additional articles.

### Eligibility criteria

#### Inclusion criteria

We included the following types of primary studies: cross-sectional, case–control, and cohort studies that reported the prevalence of CI among patients with diabetes; peer-reviewed studies published in English; studies conducted inside Africa among patients with diabetes; moderately and highly qualified studies; and freely accessible studies.

#### Exclusion criteria

The review excluded studies that did not involve patients with diabetes, those that did not provide data on CI prevalence, case reports, low-quality studies, or studies that were published in languages other than English.

### Outcome of interest

The outcome of this review is the prevalence of CI and associated factors among patients with diabetes. CI was assessed using the Mini-Mental State Examination (MMSE) in the primary studies.

### Article selection and data extraction

Duplicate articles were deleted after importing all articles into the EndNote version X7 software. Then, two authors (YAF and AMZ) screened the articles critically for eligibility criteria. The corresponding author, publication year, study setting, study design, study population, sampling procedure, total sample size, response rate (participant), associated factors, and prevalence of CI among patients with diabetes were extracted by two authors (YAF and AMZ) using a standardized Microsoft Excel data extraction format. Associated factors were extracted based on the following eligibility criteria: having a similar categorization, having a similar operational definition, having been reported with a similar statistical measure (odds ratio), having a similar direction of association, and having been associated with two or more articles.

### Quality assessment

Two authors (WCT and YAF) assessed the quality of articles using tools assessing the risk of bias in prevalence studies: modification of an existing tool and evidence of interrater agreement developed by Hoy and Brooks (21). The tool consists of 10 items addressing four domains of bias plus a summary risk of bias assessment. The 10 items are representativeness, sampling frame/procedure, random selection, non-response bias, direct data collection from patients, acceptability of case definition, study tool reliability and validity, same mode of data collection, appropriate length of prevalence period, and appropriateness of numerator and denominator. Uncertain or unclear items were considered to have a high risk of bias. After summarizing the high risk of bias, the overall risk of bias was evaluated as low (≤2), moderate (3–5), and high (≥5) ( **Supplementary Material 2** ).

### Statistical analysis

After extraction, the data were imported to Stata software version 11 (Stata Corp LLC., TX, USA) for further analysis. Heterogeneity between the included articles was assessed using Cochran’s *Q* chi-square test at a significance level of less than 0.05 and inverse variance (*I*² index). Values of 0%–40%, 40%–60%, 60%–90%, and 90%–100% indicated low, medium, substantial, and high heterogeneity, respectively (22). The random-effects model, specifically the DerSimonian and Laird (D+L) pooled estimation method, was used due to the high heterogeneity among the included articles (23). Sub-group analyses and sensitivity analyses were also conducted to handle heterogeneity. Egger’s and Begg’s regression tests and visual inspection of the funnel plot were utilized to assess the evidence of publication bias.

## Results

### Study selection

**Figure 1:**
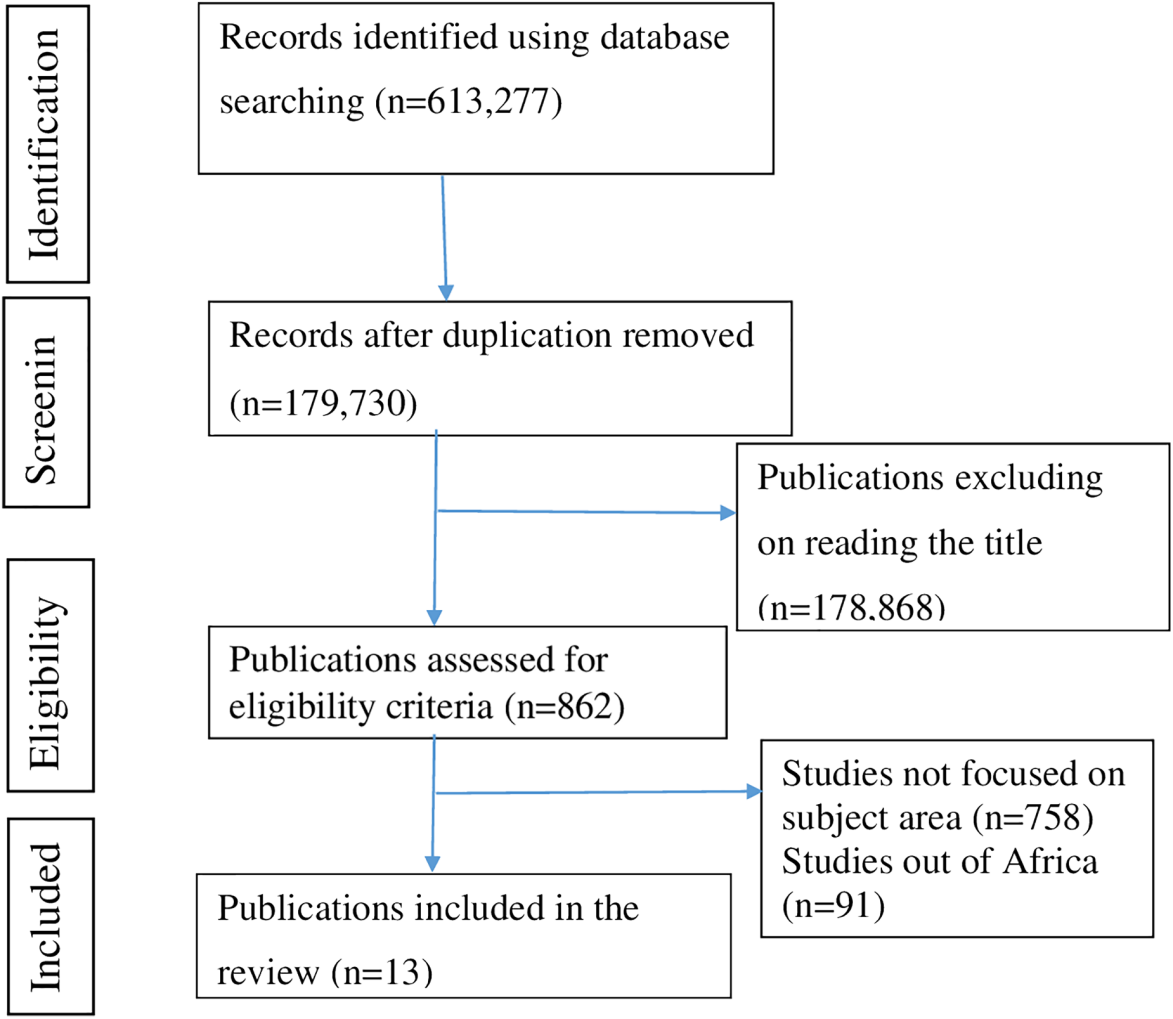
PRISMA flow diagram of the selection of publications for a systematic review and meta-analysis on the prevalence and associated factors of cognitive impairment among patients with diabetes in Africa (*N* = 13).

Our systematic search found a total of 613,277 articles from five databases: [Google Scholar (18,200), PubMed (588,989), Cochrane Library (402), Science Direct (5352), and African Journal Online (334)]. After importing all articles into Endnote, 433,547 articles were removed due to duplication. Of the remaining articles, 178,868 were excluded after title screening. The abstract text of 862 articles was assessed for eligibility criteria; finally, 13 articles ultimately met the inclusion criteria and were included in the review. A summary of the steps involved in the screening process and the reasons for the exclusion of articles is provided (**Figure 1**).

### Baseline characteristics of the included publications

The review analyzed the results of 13 articles (11 studies are institutional-based cross-sectional, and the remaining 2 are cohort and case–control studies). The included articles were conducted in different countries in Africa; three from Ethiopia (24–26), four from Nigeria (27–30), three from Egypt (31–33), and the remaining from Cameroon, Congo, and Morocco (34–36). Detailed baseline characteristics of the included articles are presented in **Table 1**.

**Table 1:** Baseline characteristics of the included articles on the prevalence and associated factors of cognitive impairment among patients with diabetes in Africa (*N* = 13).

Author	Publication year	Country	Study design	Study population	Sample size	Prevalence (%)	Sampling method
Ashebir et al. (24)	2024	Ethiopia	IBCS	Patients with DM	421	56.3	Systematic random
Mulugeta et al. (25)	2013	Ethiopia	IBCS	Patients with DM	384	45	Simple random
Abba et al. (34)	2017	Cameroon	IBCS	Patients with DM	223	14.8	Consecutive
Eze et al. (27)	2015	Nigeria	IBCS	Patients with DM	113	40	Systematic random
Abdellatif et al. (31)	2020	Egypt	IBCS	Patients with DM	200	34	Consecutive
Williams et al. (28)	2020	Nigeria	IBCS	Patients with DM	485	33.4	Consecutive
Dagnew et al. (26)	2017	Ethiopia	IBCS	Patients with DM	210	53.3	Consecutive
Mohamed et al. (32)	2023	Egypt	IBCS	Patients with DM	400	50	Simple random
Anwar et al. (33)	2018	Egypt	IBCS	Patients with DM	100	18	Consecutive
Bashir and Yarube (29)	2022	Nigeria	IBCS	Patients with DM	93	88.5	Systematic random
Ossou et al. (35)	2019	Congo	Case control	Patients with DM	200	57	Consecutive
Tlemcani et al. (36)	2022	Morocco	Cohort	Patients with DM	100	47.5	Consecutive
Adebayo et al. (30)	2022	Nigeria	IBCS	Patients with DM	274	27	Systematic random

*IBCS, institutional-based cross-sectional study.

### Quality of the included studies

Based on the quality assessment results, 11 studies (84.61%) of the included articles have a low risk of bias, and 2 have a moderate risk of bias. The detailed results of the quality assessment of the articles are provided in the **Supplementary File** (**Supplementary File 2**).

### Publication bias

**Figure 2:**
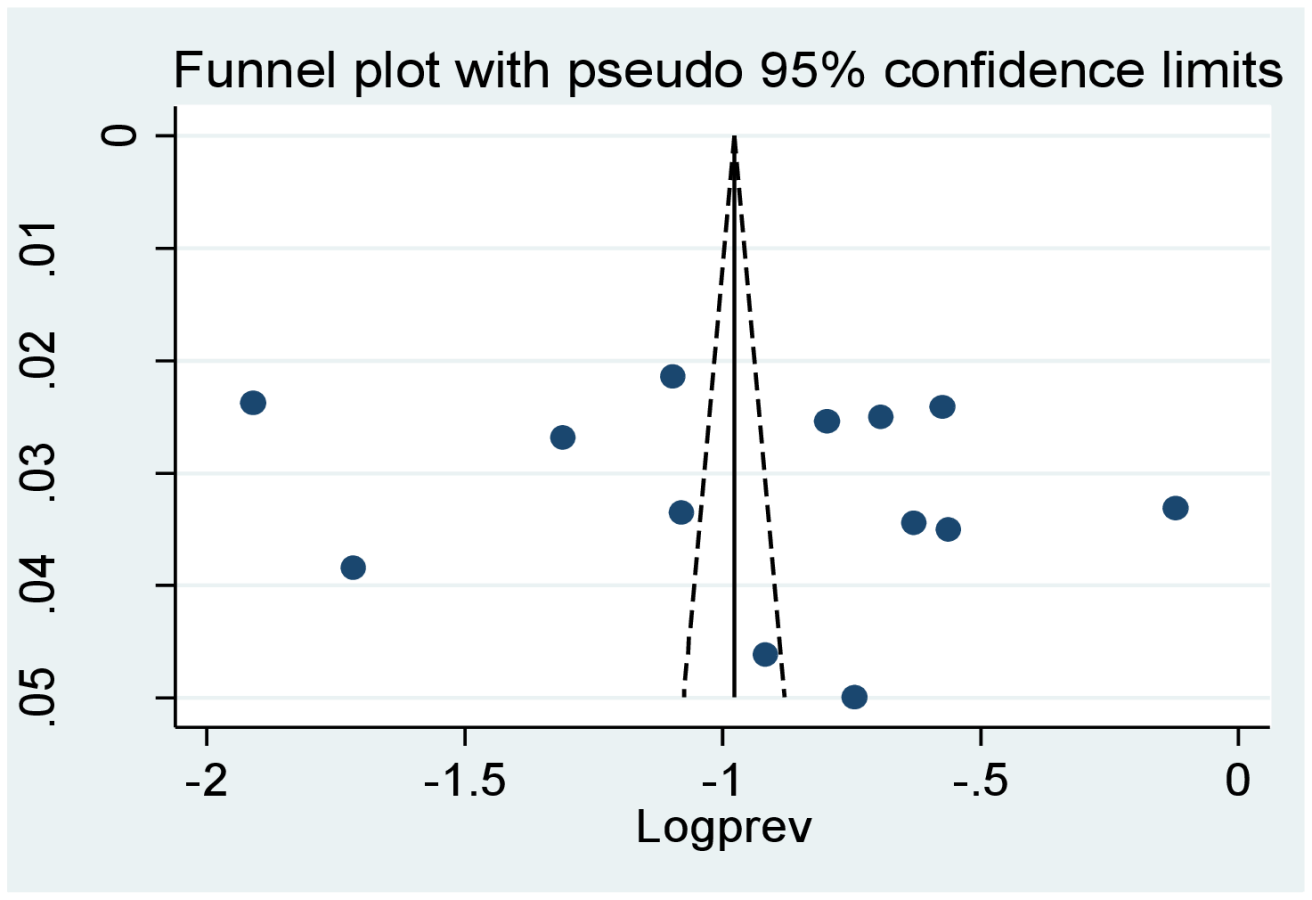
Funnel plot showing the absence of publication bias in the systematic review and meta-analysis of prevalence and associated factors of cognitive impairment among patients with diabetes in Africa (*N* = 13).

Begg’s and Egger’s regression tests were used to determine the evidence of publication bias. Based on our results, there is no significant publication bias indicated with Egger’s regression test *p*-value >0.05 (*p* = 0.532) and symmetrical inspection of the funnel plot (**Figure 2**).

### Sub-group analysis

**Figure 3:**
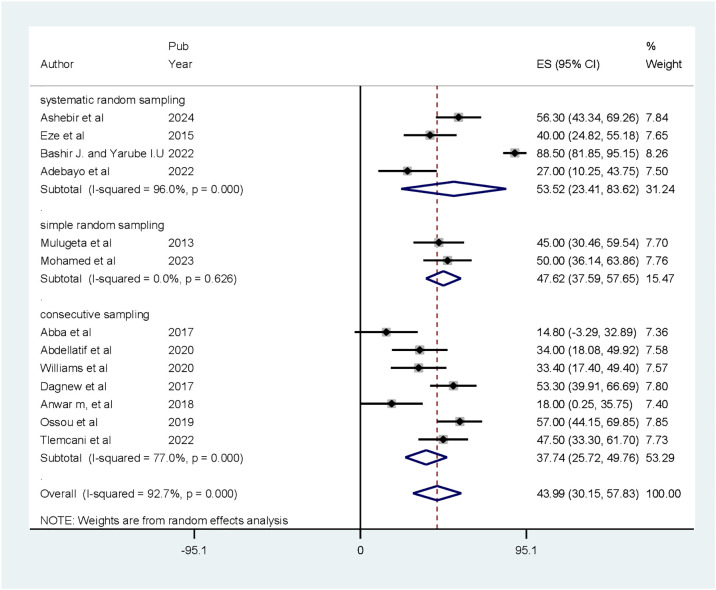
Forest plot of the sub-group analysis showing the prevalence of cognitive impairment among patients with diabetes based on sampling technique in Africa (*N* = 13).

Sub-group analysis was done based on sampling techniques. The results showed that the highest prevalence of CI was reported in articles that used systematic random sampling [53.52% (CI: 23.41, 83.62)] and there was no heterogeneity in articles that used simple random sampling (**Figure 3**).

### Meta-analysis

**Figure 4:**
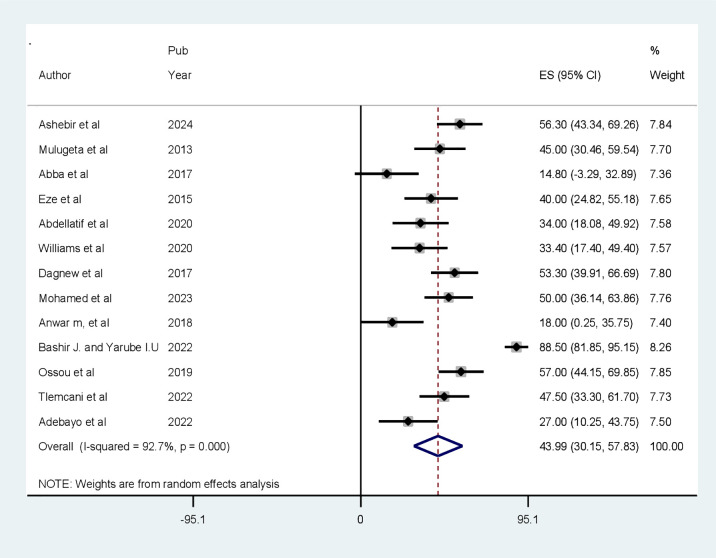
Forest plot showing the prevalence of cognitive impairment among patients with diabetes in Africa (*N* = 13).

The pooled prevalence of CI among patients with diabetes in Africa is found to be 43.99% (95% CI: 30.15–57.83, *p* < 0.001). The analysis showed a high heterogeneity between the included articles (*I* ^2^ = 92.7%, *p* < 0.001). As a result, a random-effects model, specifically the DerSimonian and Laird (D+L) pooled estimation method, was used to estimate the pooled prevalence of CI (**Figure 4**).

### Sensitivity analysis

**Figure 5:**
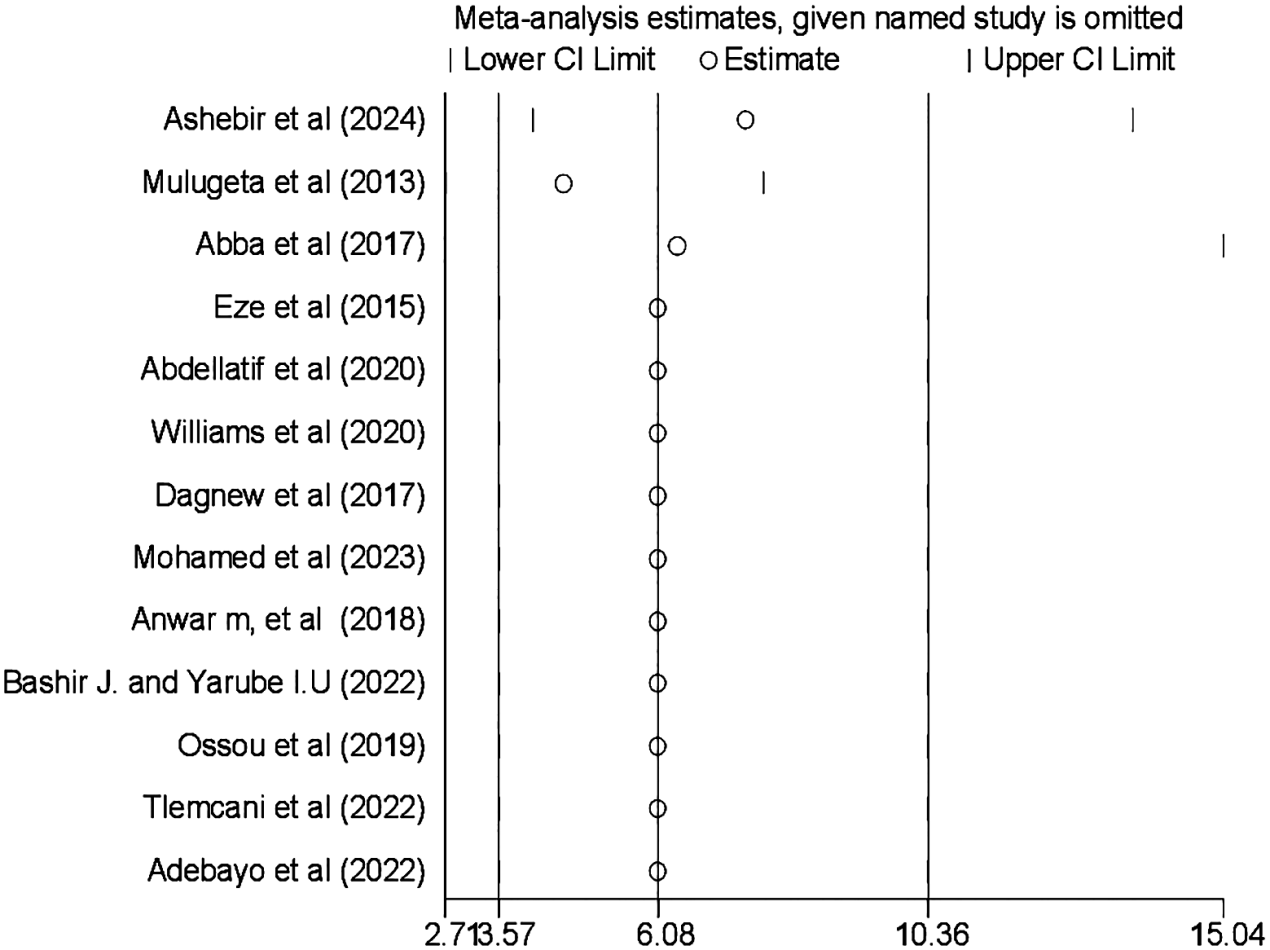
Result of the sensitivity analysis on the prevalence and associated factors of cognitive impairment among patients with diabetes in Africa (*N* = 13).

Sensitivity analysis was also conducted using the random-effects model, and the results showed that no single study influenced the pooled prevalence of CI among patients with diabetes (**Figure 5**).

### Associated factors of cognitive impairment

**Figure 6:**
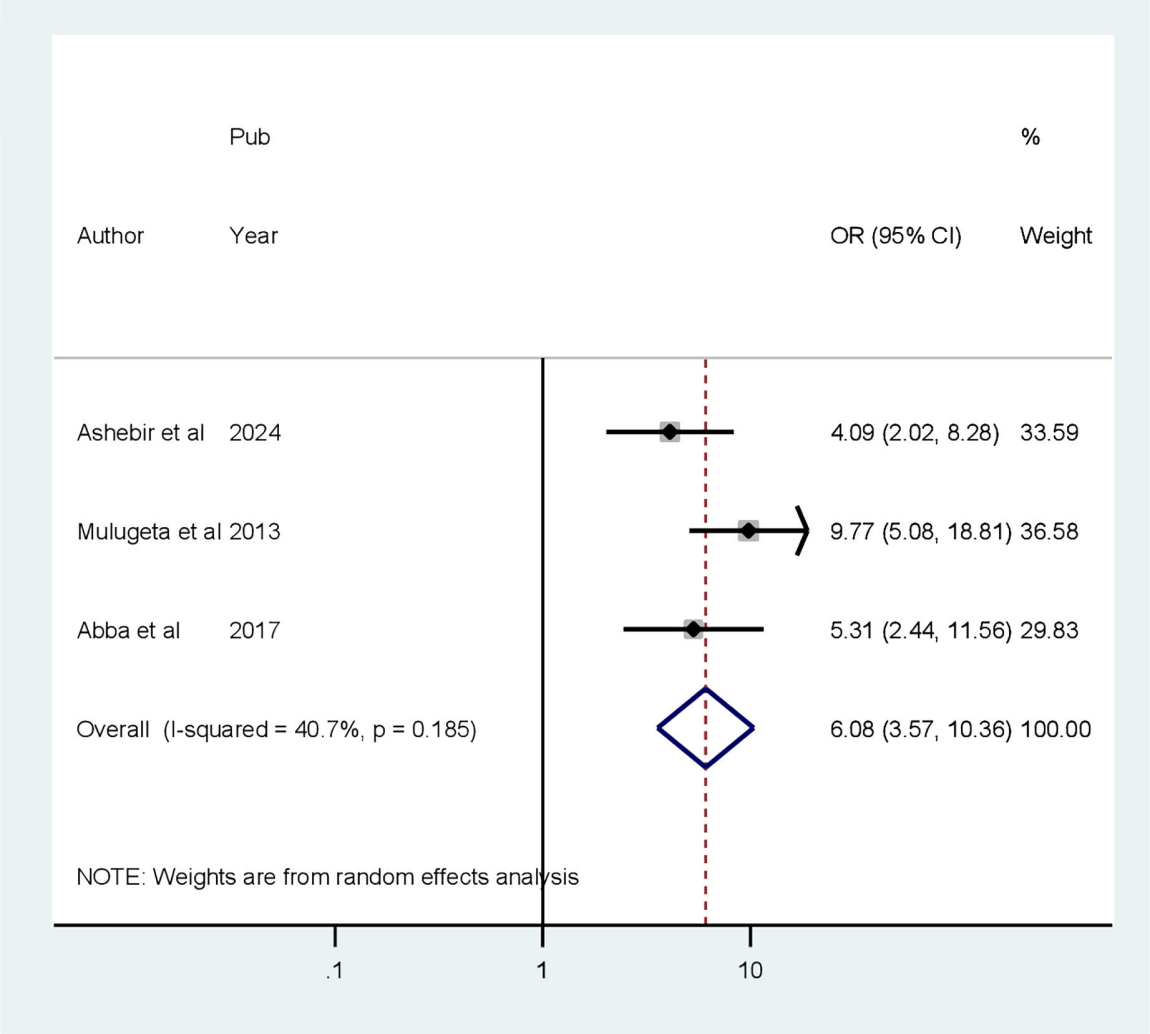
Forest plot showing the association between primary education level and cognitive impairment among patients with diabetes in Africa (*N* = 13).

**Figure 7:**
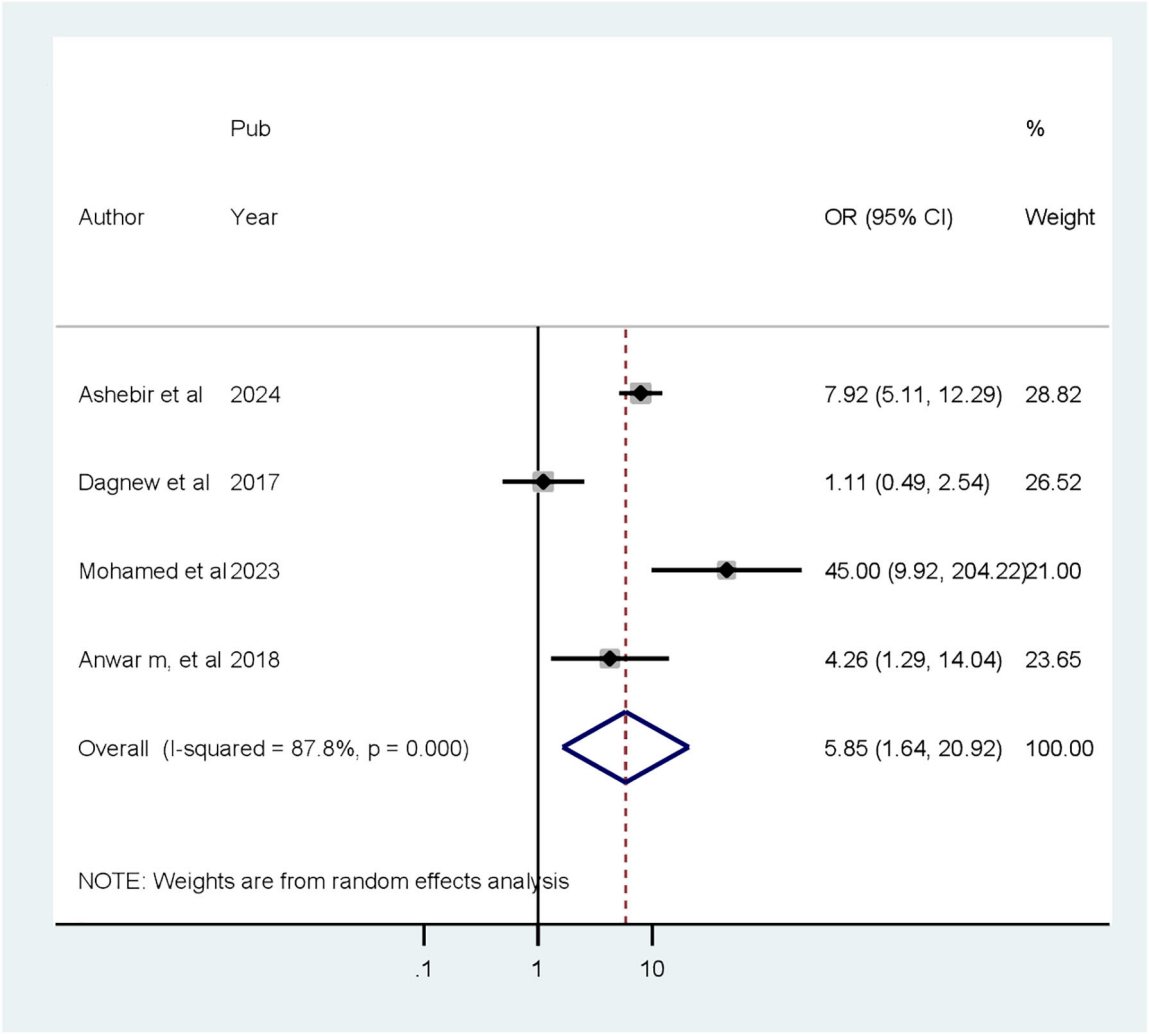
Forest plot showing the association between uncontrolled DM and cognitive impairment among patients with diabetes in Africa (*N* = 13).

**Figure 8:**
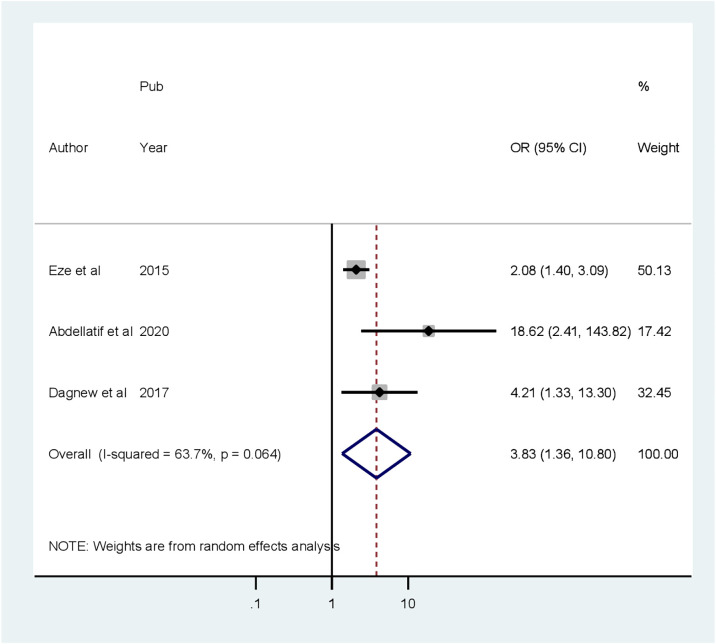
Forest plot showing the association between age greater than 60 years old and cognitive impairment among patients with diabetes in Africa (*N* = 13).

**Figure 9:**
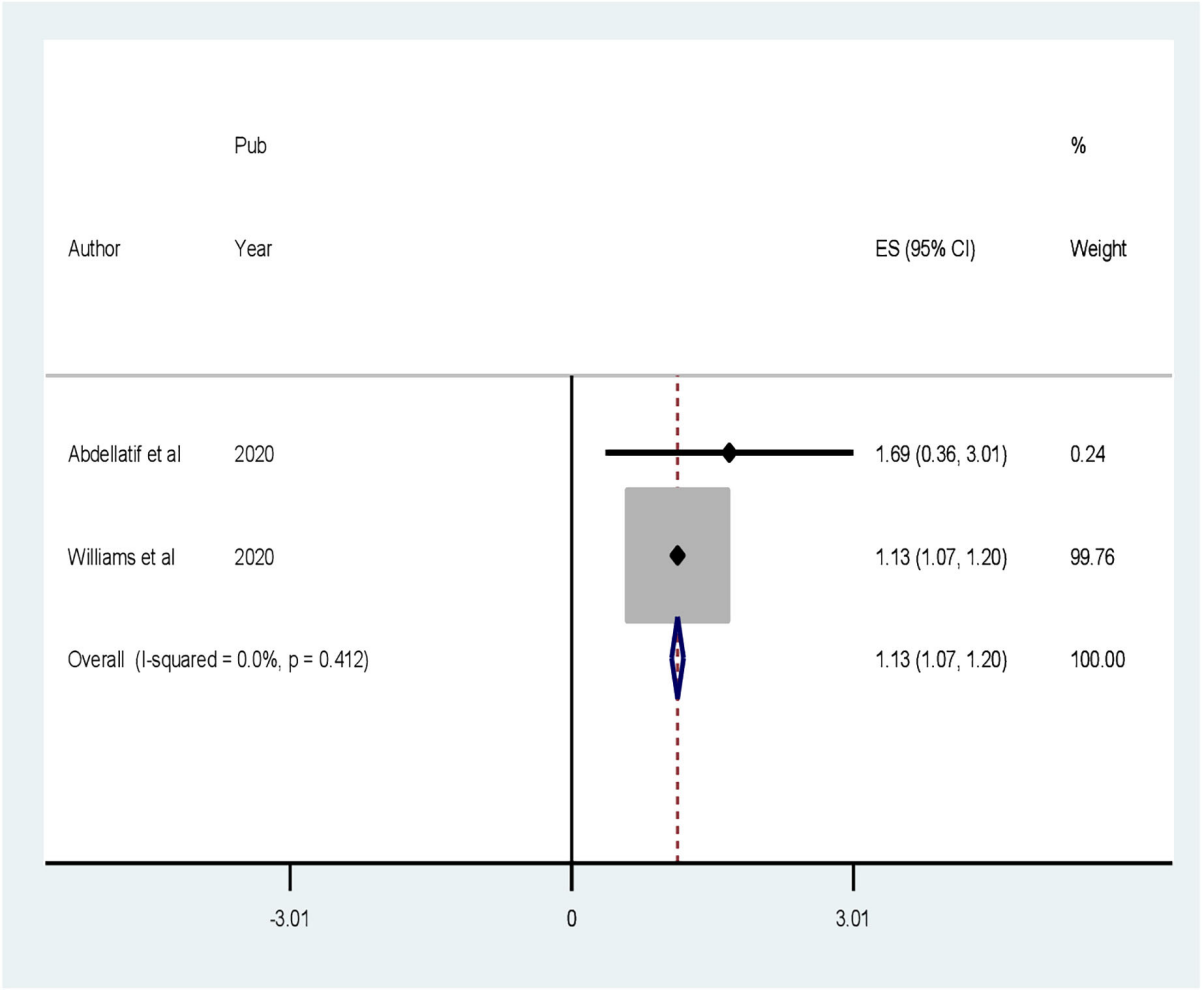
Forest plot showing the association between the duration of DM greater than 10 years and cognitive impairment among patients with diabetes in Africa (*N* = 13).

Four factors are associated with CI among patients with diabetes, based on extracted factors from the primary articles. They are primary educational level, uncontrolled DM, age greater than 60 years old, and duration of DM greater than 10 years. According to our analysis, the random pooled odds ratio of developing CI was 6.08 times (POR = 5.85, 95% CI: 1.64–20.92, *I* ^2^ = 87.8%) higher among patients with diabetes who completed primary education compared to college and post-college education(**Figure 6**). The random pooled odds ratio of developing CI was 5.85 times higher (POR = 3.83, 95% CI: 1.36–10.79, *I* ^2^ = 63.7%) among patients with DM whose blood glucose was uncontrolled as compared to patients with DM whose blood sugar was controlled (**Figure 7**). Patients with diabetes whose age was above 60 years old were 3.83 times more likely to develop CI (POR: 1.13; 95% CI: 1.07–1.19, *I* ^2^ = 0.0%) than patients whose age was lower than or equal to 60 years old (**Figure 8**). The review also found that patients with DM who had diabetes for more than 10 years were 1.13 times more likely to develop CI (POR: 1.13; 95% CI: 1.07–1.19, *I* ^2^ = 0.0%) than patients with DM for less than 10 years (**Figure 9**).

## Discussion

According to the review, the pooled prevalence of CI among patients with diabetes in Africa is found to be 43.99% (95% CI: 30.15–57.82, *p* < 0.001). This result is in line with the findings of studies conducted in China, 45.0% (95% CI: 36.0–54.0) (13); India, 33.73% (37); Korea, 31.5% (38); Philippines, 45% (39); and Poland, 32.7% (40).

However, the finding is higher than the result of studies done in Japan, 26% (41); China, 21.8% (14) and China, 13.5% (42); USA, 25.6% (43); and New York, 28% (44). The elevated prevalence observed in our review could stem from several factors. First, in the setting of the aforementioned study, hospitalized patients may typically experience better plasma glucose management as physicians prioritize close monitoring of their levels, potentially leading to a lower prevalence in this group. Conversely, in our study setting, the study population tends to be of lower socioeconomic status, which is linked to poorer cognitive function due to limited resources and healthcare access. Additionally, variations in the study populations, such as the inclusion of individuals with advanced DM in the Nigerian study, may significantly contribute to the occurrence of complications and cognitive decline. Finally, the substandard quality of healthcare services in African contexts may also play a role in the higher prevalence observed. The other interesting reason for this discrepancy is that carriers of apolipoprotein E (*APOE*) ϵ4 are at high risk for cognitive decline and Alzheimer’s disease. The ϵ4 allele is more common in Black than white individuals. The ϵ4 allele is associated with a risk of cognitive decline and dementia (45).

However, the pooled prevalence of CI in our review is lower than the findings of studies done in India, 58.29% (46) and 64.86% (47) and Pakistan, 67.3% (15). A potential explanation could be the variation in the age demographics of the subjects studied. The research conducted in Pakistan involved older patients with DM, who are more prone to age-related cognitive decline. Conversely, studies in India focused on patients with chronic DM, who are at higher risk for various complications, including CI.

According to our analysis, the random pooled odds ratio of CI among patients with diabetes who completed primary education was 6.08 times higher as compared to those whose educational level was college or above. The finding is similar to the results of studies conducted in China and Germany (48, 49). This may be due to the fact that individuals with higher education tend to have a larger “cognitive reserve,” meaning that they have a greater capacity for mental processing and can better compensate for age-related declines in brain function. This reserve may be built through years of stimulating mental activity, including learning new information, problem solving, and engaging in complex tasks.

The random pooled odds ratio of developing CI among patients with DM whose blood glucose was uncontrolled was 5.85 times higher compared to patients with DM whose blood sugar was controlled. The finding is consistent with the results of studies done in New York and India (38, 50). This is explained by the fact that elevated blood sugar levels in diabetes can directly damage neurons through an increased polyol pathway, advanced glycation end products, protein kinase C (PKC) activation, and increased production of free radicals (highly reactive molecules that damage cells and DNA) (51).

Patients with diabetes whose age was above 60 years old were 3.83 times more likely to develop CI than patients whose age was lower than or equal to 60 years old. This is in line with the findings of a study done in India (52). The possible justification for this might be that as age increases, the brains naturally undergo changes that can contribute to cognitive decline, including neuronal loss (gradual death of neurons throughout the brain, particularly in areas critical for memory, learning, and reasoning), synaptic dysfunction (weakening of connections between neurons, hindering communication and information processing), and neuro-inflammation (chronic low-grade inflammation in the brain, which can damage neurons and impair cognitive function) (48). The other justification for this may be due to a decline in processing speed, working memory, and episodic memory (recalling specific events) with age (52). Another reason for this may be due to early-onset familial Alzheimer’s disease (EOFAD), which has some distinctive features including early age at onset, positive family history, a variety of non-cognitive neurological symptoms and signs, and a more aggressive course. There is marked phenotypic heterogeneity among different mutations in EOFAD.

The review also identified that patients with DM who had diabetes for more than 10 years were more than 1.13 times more likely to develop CI than patients with diabetes duration of less than 10 years. The finding is in line with the studies done in China, Mexico, and London (42, 53, 54). This is justified by the long duration of DM as an atherogenic factor; it may increase the risk of cognitive dysfunction through well-recognized associations with stroke, causing cerebral disease and cerebral infarction (55).

### Limitations

This review has some limitations. The sub-group analysis did not indicate adequate factors to explain the observed high heterogeneity; important factors associated with CI were not analyzed in our review because of the lack of information in the primary studies and inconsistent categorization, and the number of included articles was not adequate (*N* = 13), which may affect the representativeness of the results.

## Conclusion and recommendation

Based on our systematic review, individuals with DM exhibit a substantial prevalence rate (43.99%) of CI. CI was found to be associated with factors such as primary level of education, poorly controlled DM, age above 60 years, and diabetes duration greater than 10 years. Developing suitable risk assessment tools is crucial to address uncontrolled hyperglycemia effectively. Healthcare professionals in Africa ought to prioritize the monitoring of cognitive function in patients with DM. Early identification of CI among patients with DM is beneficial for both restoring cognitive function and slowing down cognitive decline. Healthcare institutions need to create diagnostic and treatment plans tailored to individuals with chronic diabetes to address cognitive issues, with a particular emphasis on the elderly population. Additional interventional research endeavors focused on mitigating cognitive decline in DM, particularly those targeting novel risk factors in primary care settings, are recommended.

## Data availability statement

The original contributions presented in the study are included in the article/**Supplementary Material**. Further inquiries can be directed to the corresponding author.

## Author contributions

WC: Writing – review & editing, Writing – original draft, Visualization, Validation, Supervision, Software, Resources, Project administration, Methodology, Investigation, Funding acquisition, Formal analysis, Data curation, Conceptualization. YF: Data curation, Writing – original draft. AZ: Data curation, Writing – original draft.
